# Advances in Stroke Neurorehabilitation

**DOI:** 10.3390/jcm12216734

**Published:** 2023-10-25

**Authors:** Muhammed Enes Gunduz, Bilal Bucak, Zafer Keser

**Affiliations:** 1 Department of Neurology, University of Massachusetts Chan Medical School, Worcester, MA 01655, USA; 2 Department of Neurology, Mayo Clinic, Rochester, MN 55905, USA; bucak.bilal@mayo.edu (B.B.); keser.zafer@mayo.edu (Z.K.)

**Keywords:** stroke, neurorehabilitation, neuromodulation, brain computer interfaces, virtual reality

## Abstract

Stroke is one of the leading causes of disability worldwide despite recent advances in hyperacute interventions to lessen the initial impact of stroke. Stroke recovery therapies are crucial in reducing the long-term disability burden after stroke. Stroke recovery treatment options have rapidly expanded within the last decade, and we are in the dawn of an exciting era of multimodal therapeutic approaches to improve post-stroke recovery. In this narrative review, we highlighted various promising advances in treatment and technologies targeting stroke rehabilitation, including activity-based therapies, non-invasive and minimally invasive brain stimulation techniques, robotics-assisted therapies, brain–computer interfaces, pharmacological treatments, and cognitive therapies. These new therapies are targeted to enhance neural plasticity as well as provide an adequate dose of rehabilitation and improve adherence and participation. Novel activity-based therapies and telerehabilitation are promising tools to improve accessibility and provide adequate dosing. Multidisciplinary treatment models are crucial for post-stroke neurorehabilitation, and further adjuvant treatments with brain stimulation techniques and pharmacological agents should be considered to maximize the recovery. Among many challenges in the field, the heterogeneity of patients included in the study and the mixed methodologies and results across small-scale studies are the cardinal ones. Biomarker-driven individualized approaches will move the field forward, and so will large-scale clinical trials with a well-targeted patient population.

## 1. Introduction

Stroke is one of the leading causes of long-term disability worldwide. Stroke rehabilitation therapies aim to enhance favorable neuroplasticity. With the advances in technology, the stroke recovery field is rapidly evolving and various promising novel treatment approaches and technological advances are becoming part of the therapeutic armamentarium to improve post-stroke recovery in both the post-acute and chronic phases. In order to inform and guide medical providers treating patients with stroke, this review aims to discuss these advances, including activity-based therapies, technology-assisted therapies, non-invasive and minimally invasive brain stimulation techniques, lesion bypass systems, pharmacological treatments, and cognitive therapies. 

## 2. Non-Invasive/Minimally Invasive Brain Stimulation

**Figure 1:**
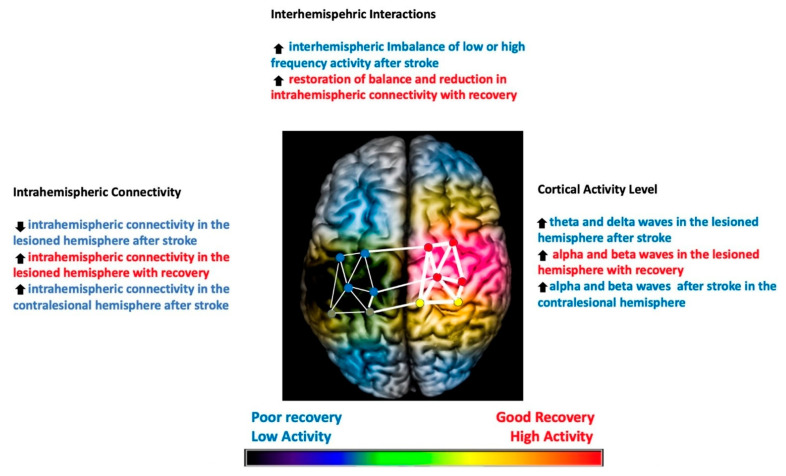
Illustration of the post-stroke EEG changes in the brain and patterns of recovery identified on the EEG correlating with good (highlighted in red) and poor recovery (highlighted in blue). Degree of coherence of intrahemispheric connectivity is represented by the width of the lines in the network. The overall activity of the hemisphere, as well as nodes in the network, is represented in a color-coded manner (red: most active, yellow: less active, blue: least active, no color: no activity). Stroke infarct is represented as a dark shade on the lesioned hemisphere. Up arrow means increased and down arrow means decreased.

Recovery from stroke requires extensive plastic changes in the nervous system and their adaptation. The initial spontaneous recovery occurs via this neuroplasticity, mainly by neuroregeneration and reorganization. Functional magnetic resonance imaging (fMRI) studies showed reorganization of the lesional area into the non-lesional cortex [1,2]. This reorganization is associated with increased bilateral activation in the early/acute phase, which becomes lateralized towards the non-lesional hemisphere in the chronic phase. The later changes typically lead to a maladaptive interhemispheric imbalance, which may interfere with recovery. These post-stroke intra and interhemispheric changes were further characterized by electroencephalogram (EEG) and used to understand mechanisms of recovery and treatment responses. After stroke, the higher frequency activities (≥8 Hz, such as alpha and beta waves) and intra-hemispheric connectivity are reduced, which results in imbalanced activity between lesioned and non-lesioned hemispheres [3]. The compensatory changes in the non-lesioned hemisphere discussed above are hypothesized to worsen this interhemispheric imbalance. Therefore, the recovery mechanisms are focused on restoring interhemispheric balance and increasing intrahemispheric connectivity in the lesioned hemisphere. Figure 1 illustrates these post-stroke changes and recovery patterns. 

The concept of brain stimulation techniques aims to modulate neural networks specifically and selectively by enhancing adaptive patterns and suppressing maladaptive ones [4]. The historical use of brain stimulation dates as early as the 11th century, and in the 1960s, scientific studies on animal models using weak electric current to the exposed motor cortex showed long-lasting polarizing effects [5] and subsequently in human studies [6]. The hypothetical model of brain stimulation modulated the imbalanced interhemispheric inhibition by activating the lesional/affected hemisphere while inhibiting the non-lesional/unaffected hemisphere. The rationale of brain stimulation techniques is to improve recovery by enhancing adaptive plasticity when applied concomitantly with other therapies. The application of brain stimulation techniques has increased significantly, and we will focus on current advances in non-invasive/minimally invasive stimulation techniques, particularly vagus nerve stimulation (VNS), transcranial magnetic stimulation (TMS), and transcranial direct current stimulation (tDCS). Table 1 summarizes prominent recent clinical trials in neuromodulation.

### 2.1. Vagus Nerve Stimulation

Vagus nerve stimulation (VNS) is a minimally invasive neuromodulation technique provided concomitantly with behavioral therapies such as task-oriented arm therapy in stroke to enhance motor recovery. Meyer and colleagues provided an initial proof of concept that, compared to sham stimulation, coupling VNS with rehabilitative training resulted in a twofold increase in long-term recovery for complex and basic motor tasks in rats with stroke [12]. Overall, the proposed mechanism of VNS in post-stroke motor recovery is the reorganization of the primary motor cortex through modulation of cholinergic and noradrenergic retrograde projections of nucleus tractus solitarius (one of the main vagus nuclei). 

Initial clinical studies also showed that when combined with task-oriented upper limb therapy, VNS was shown to increase motor recovery in patients with chronic stroke [13,14]. This led to a phase III pivotal trial in which Dawson and colleagues performed a randomized, sham-controlled, blinded, adequately powered study that showed that compared to sham stimulation, patients in the active VNS groups had a significantly higher likelihood of achieving clinically meaningful motor recovery (measured by at least six points increase in the upper extremity Fugl-Meyer assessment) at 90 days [7]. After the positive results of the pivotal phase III trial, the United States Food and Drug Administration (FDA) approved the use of VNS in patients with chronic ischemic stroke and moderate to severe arm weakness.

The real-world implementation of VNS for post-stroke upper extremity recovery is still underway. A multidisciplinary approach, including vascular neurology, neurosurgery, and rehabilitation medicine, is crucial for success. Additionally, patient compliance and the financial burden for long-term management are other critical factors for real-world implementation. Finally, stroke patients generally have multiple comorbidities potentially requiring antithrombotic use, which might make them ineligible for the procedure. Although non-invasive VNS (nVNS) might be an alternative treatment for patients ineligible for VNS therapy, previous studies failed to show consistent neuromodulatory effects of nVNS in the motor cortex [15,16].

Additionally, further investigation should be pursued as to whether VNS will show similar benefits for other post-stroke deficits such as aphasia, cognitive impairment, and dysphagia. The safety and efficacy of VNS in hemorrhagic or subacute stroke are other frontiers in VNS research [17].

### 2.2. Transcranial Magnetic Stimulation

TMS modulates cortical excitability through the scalp via coil using the principle of electromagnetic induction. This electromagnetic field induces a focal electric current in the brain sufficient to depolarize neurons transiently. Single or paired-pulse TMS is commonly used as an assessment tool to explore brain functioning, while when applied repetitively—repetitive TMS (rTMS)—they can modulate cortical excitability in a sustained manner. This modulation of excitability depends on the parameters of stimulation; high-frequency (≥5 Hz) rTMS induces excitatory effects, while low-frequency (≤1 Hz) induces inhibitory effects. rTMS is an FDA-approved treatment for multiple psychiatric disorders such as major depression, obsessive compulsive disorders, and migraines. rTMS is considered safe (only non-serious adverse effects) when appropriate protocol and screening are used [18].

The effects of both high- and low-frequency rTMS on motor recovery have been studied in over 70 studies; most studies focus on upper extremity impairment, with limited data available for the lower extremity [19,20]. The meta-analyses and evidence-based guidelines suggest that using low-frequency over the contralesional hemisphere and high-frequency rTMS over the ipsilesional hemisphere in the subacute phase is potentially effective. At the same time, very limited evidence exists for the use of rTMS in the chronic phase to enhance upper extremity rehabilitation [21]. However, current evidence from randomized controlled trials is conflicting. An important phase III, multicenter, double-blinded, randomized, sham-controlled trial assessed 18 sessions of low-frequency rTMS over the motor cortex combined with intensive goal-oriented motor training in chronic stroke patients with moderate upper extremity hemiparesis [9]. Although both groups showed significant clinical improvement with intense therapy, additional rTMS did not offer other benefits. These results were similar to their recent extension study (E-FIT: Electric Field Navigated 1-Hz rTMS for Poststroke Motor Recovery) designed to address concerns in sham TMS coil in the previous study [22]. Another group used a similar design in patients within the first three months after stroke; similarly, there was no significant difference between the active and sham rTMS groups [23].

On the other hand, a randomized sham-controlled clinical trial recently showed that contralateral continuous theta burst stimulation (inhibitory) over the contralesional hemisphere could improve upper limb recovery in stroke patients within 3 weeks of symptom onset [10]. All these conflicting results from various studies suggest that the theory of inhibitory stimulation over the contralateral motor cortex is considered simplistic, given the heterogeneity of strokes involving motor deficits. Further evidence suggests that the structural reserve after stroke contributes to recovery models (bimodal balance–recovery model). Therefore, future studies need to consider lesion anatomy, the extent of damage to transcallosal networks, and the integrity of the corticospinal tract for better patient stratification [24].

Aphasia recovery is also studied using rTMS based on the same interhemispheric inhibition hypothesis. The literature suggests beneficial perilesional activation in chronic aphasia patients; however, the role of right hemisphere activation is unclear. Interestingly, an fMRI study showed a robust right-sided contralesional activation selectively during incorrect naming responses in post-stroke aphasia patients compared to healthy subjects, which may reflect a dysfunctional reorganization rather than compensatory [25]. A diffusion tensor imaging study further supported this [26]. Therefore, most rTMS studies focused on delivering low-frequency inhibitory stimuli over the right hemisphere in combination with traditional language therapies. While the initial studies on heterogenous patient population/aphasia type with variable protocols had mixed results, recent studies on non-fluent aphasia, especially Broca’s aphasia at the chronic stage, showed promising effects of low-frequency rTMS over the right inferior frontal gyrus [27]. A phase II, randomized, controlled, blinded trial, is in the recruitment phase to further test this in combination with constraint-induced language therapy (NCT03651700).

Hemispatial neglect, a common and disabling condition, is described as pathologically asymmetric spatial performance, particularly in strokes involving the right posterior parietal and superior temporal gyri. Based on initial evidence of rTMS effects on reducing contralesional extinction by Oliveri and colleagues, studies focused on excitability-reducing paradigms such as low-frequency rTMS and continuous theta burst stimulation (cTBS) over the contralesional-hemisphere [28,29]. Despite studies suggesting improvement with rTMS over the contralesional hemisphere, the evidence is limited given most of these studies were not controlled. Also, another sham-controlled study compared low-frequency rTMS of the contralesional hemisphere, high-frequency rTMS of the ipsilesional hemisphere, and sham stimulation. It showed better improvement of visuospatial neglect in ipsilesional high-frequency rTMS compared to other groups [30]. cTBS, another inhibitory TMS stimulation protocol, has more promising evidence in visuospatial neglect. Five sham-controlled studies with overall similar protocols showed significant clinical improvement in cTBS over the left posterior parietal cortex, as well as improvement in global functioning [21,31,32,33,34]. A recent study showed the response to cTBS was associated with the integrity of interhemispheric connections within the corpus callosum, indicating that responders had corpus callosum intact but not in non-responders [33]. Currently, Level C evidence supports the use of cTBS of the contralesional hemisphere for visuospatial neglect [21]. Further studies are needed to test other visual-cognitive disorders with different pathophysiologies and establish these neuroanatomical markers to identify responders.

### 2.3. Transcranial Direct Current Stimulation

tDCS consists of applying low-amplitude, steady, direct current via electrodes placed over the skull. The current from the electrode sufficiently penetrates the underlying brain tissue by flowing across the scalp. It provides a sub-threshold stimulus that modulates neuronal transmembrane potentials and, therefore, influences the level of polarization and likelihood of neuronal firing [35]. Two modes of tDCS have been most commonly used: anodal stimulation increases excitability and cathodal stimulation decreases the excitability of the cortex. The prolonged clinical effects are attributed to long-term potentiation and depression if applied for a sufficient duration and intensity [36]. 

tDCS stimulates a wider area of the cortex in a less targeted fashion compared to techniques like TMS, which can be advantageous by stimulating additional regions that may play a role in recovery. Additionally, it has further advantages such as a better safety profile and ease of use. Important parameters in tDCS studies include the location of electrodes (montage/stimulation target), electrode sizes (current density), current intensity, duration, and frequency. No irreversible severe side effects were seen with tDCS when appropriately screened; common side effects include mild, temporary headache, skin irritation/redness, and tingling/itching sensation during dose ramp up/down [37].

Motor recovery is the most studied in tDCS studies, modulating interhemispheric inhibition by increasing excitability in the lesional hemisphere or decreasing excitability in the non-lesional hemisphere. The proof-of-concept studies showed improvement in upper extremity motor function, assessed mainly by the Fugl-Meyer scale, which could last for several weeks and was associated with increased cortical excitability in the ipsilesional hemisphere. Most clinical trials combined tDCS with various therapies, including occupational/physical therapy, constraint-induced movement therapy (CIMT), robotic-assisted therapy, or virtual reality. Unfortunately, these trials showed mixed results and effect sizes in post-stroke motor recovery, particularly given the significant variety in stimulation parameters and study designs. A meta-analysis showed a dose–response relationship in motor recovery; higher charge and current densities correlated with higher clinical improvement [38]. Furthermore, the effect size was larger in chronic stroke patients and with bihemispheric montage. Currently, TRANSPORT2, a multicentered, randomized, triple-blinded, phase 2 clinical trial is investigating the effects of tDCS at different dosage levels combined with an efficacy-proven rehabilitation therapy (CIMT) (NCT03826030). The current literature suggests a possible role of tDCS as an adjuvant therapy for post-stroke upper extremity motor recovery, particularly when applied in chronic stroke patients. Further directions include developing biomarkers such as imaging biomarkers assessing the structural and functional integrity of descending motor tracts [39].

Aphasia recovery is another potential use of tDCS as an adjunctive therapy to improve the effects of aphasia treatments. Based on several promising pilot studies [40,41,42,43], a double-blinded, randomized, controlled study assessed the effects of three weeks of fMRI-guided anodal tDCS over the language cortex during outpatient speech therapy in 74 patients with post-stroke aphasia with varying types of aphasia (Broca’s aphasia being the most common type) [8]. This study, with a futility design, showed the feasibility and possible clinical improvement in speech production (naming). Most of these studies were conducted on chronic stroke patients; therefore, another recent trial tested the effects of MRI-guided anodal tDCS over the language cortex combined with computer-delivered language therapy in subacute poststroke aphasia patients (varying types of aphasia included in the study) [11]. This study showed no significant difference in naming performance one week after therapy, likely due to a higher variability of improvement in this subacute stroke population. Interestingly, in their secondary analysis, the discourse significantly improved in the tDCS group five weeks after therapy, which is critical to life participation and quality of life. All these studies showed favorable safety profiles, and there were no detrimental effects of stimulating over longer periods, even up to 20 weeks. These two studies underline the importance of using MRI guidance in individualizing the treatment targets across the subjects. Also, since they determined the stimulation target based on fMRI or MRI, they liberally included different types of aphasia rather than creating aphasia type-based enrollment criteria and treatment protocols. However, non-fluent aphasia is typically the most common type of aphasia included in the tDCS studies [8]. A recent meta-analysis showed that anodal tDCS, particularly over the left inferior frontal gyrus (the typical location for Broca’s aphasia), is most promising among multiple different tDCS stimulation types [44]. Further confirmatory trials are needed for clinical translation, given mixed results. Further studies are currently underway with new stimulation techniques/montages to optimize the stimulation of cortical networks involved in speech. Future studies with larger sample sizes, longer and higher dose therapies, and more outcome measures are needed.

Besides tDCS, two newer electric stimulation techniques, transcranial alternating current stimulation (tACS) and transcranial random noise stimulation (tRNS), use weak alternating currents to stimulate the cortex. tACS delivers sinusoidal currents (0.1–80 Hz) between electrodes, while tRNS delivers a wider range frequency (0.1–640 Hz) in a random order, and they modulate neuronal activity by influencing brain oscillations. These techniques are more commonly tested in behavioral and movement disorders, but few proof-of-concept studies suggested feasibility and possible effects in stroke recovery, especially in the chronic phase [45,46,47]. Current evidence is limited for clinical translation; more studies are ongoing to assess the efficacy and mechanism of these alternating current stimulations in different modalities, including motor, sensory, speech, cognitive, and visual recovery (NCT04043689, NCT05576129, NCT06029062, NCT06048159, NCT05466487).

Finally, neuromodulation techniques have been used to treat disorders of consciousness, defined as an alteration in arousal and awareness. This disorder is commonly caused by cardiac arrest, traumatic brain injury (TBI), and stroke, especially intracerebral hemorrhage. Unfortunately, currently, amantadine is the only beneficial treatment shown by a randomized trial on TBI patients, despite the significant burden to patients, caregivers, and healthcare. Besides many pharmacological treatments, TMS [48,49,50,51,52,53,54,55,56,57], tDCS [58,59,60,61,62,63,64,65,66,67,68], tACS [69], tRNS [70], and VNS [71,72] are considered possible treatment approaches to restore consciousness by neural restoration across large cortical-thalamo-cortical networks. TMS and tDCS were more commonly studied, targeting either the dorsolateral prefrontal cortex or the motor cortex (M1). Despite positive results on consciousness, these studies are limited to drawing a conclusion, given most of them were case reports/case series or small sample-sized randomized studies with mixed patient populations. Neuromodulation can be a promising tool for prognostication and recovery in stroke patients with disorders of consciousness; current pilot/small studies are encouraging, and further robust large sample-sized clinical trials are needed.

## 3. Activity-Based Therapies

Activity-based therapies are the key component of stroke neurorehabilitation. The principle of these therapies is to provide structuralized activities with adequate quantity and quality to induce plasticity for recovery. However, there are many challenges in clinical and research settings, including a lack of standardization, limited dose and intensity, and variability of responders. While the therapies with favorable plasticity use high intensity in animal studies with a range of 300–800 repetitions, achieving similar intensity in clinical settings has been challenging [73,74]. A study observed in in-clinic therapies showed a mean of 32 repetitions per session, which was a substantially smaller dose compared to animal studies [75]. We will highlight current advances in promising therapies aiming to provide higher quality and quantity of rehabilitation. Table 2 summarizes prominent recent clinical trials in activity-based therapies.

### 3.1. Constraint Induced Movement Therapy

Constraint-Induced Movement Therapy (CIMT) is an effective and popular rehabilitation approach primarily designed to improve the functional use of an affected limb in stroke patients. This therapy involves an intense functionally oriented task practice of the paretic extremity along with restraint of the less-impaired extremity for most waking hours [79]. The main two principles of CIMT are (i) constraint of forced use of the less-affected limb with a splint or mitt, preventing its use during 90% of the day and therefore promoting more frequent and intensive use of the more impaired limb; (ii) intensive training of more affected limb with task-oriented, structured, and repetitive activities for up to 6 h a day for two weeks.

The history of constraint-induced therapy theory dates back to the early 1900s when the phenomena of motor disability due to disuse was described in monkeys with pyramidal tract lesions by Franz and colleagues [80]. Subsequently, they provided the first documented evidence that these monkeys, forced to use their hemiparetic extremity by immobilizing the better limb, had increased and faster recovery. It was hypothesized that the deafferentation leads to inactivity and behavioral changes of not using the affected limb; “learned nonuse” could be overcome by behavioral strategies such as CIMT [81]. The current evidence is from the multicentered, randomized, single-blinded EXCITE trial, the first National Institute of Health (NIH)-funded stroke neurorehabilitation trial, and the only randomized-controlled Phase III trial that has shown efficacy in stroke recovery [76]. EXCITE trial showed significant and clinically relevant improvement in upper extremity motor function in patients with first stroke within the previous 3–9 months as a result of 10 days of CIMT therapy. These clinical improvements persisted for at least one year after the intervention. Although this therapy was designed to improve upper extremity weakness, further studies in the lower extremity suggested improvement in motor functions and balance-related motor function [82]. The current evidence supports the use of CIMT in stroke patients, particularly in upper extremity motor recovery.

Besides the theory of overcoming “learned nonuse”, plasticity plays an important role in CIMT outcomes. Several studies showed cortical reorganization after CIMT-based interventions by changes in the size and excitability of regions representing the affected limb [83,84]. Researchers evaluated the motor cortex changes with TMS motor mapping and showed an increase in the size of hand muscles only in the ipsilesional hemisphere after CIMT treatment, as well as changes in excitability corresponding to recovery [85]. Given these changes in inter and intra-cortical excitability, further investigations of CIMT in combination with non-invasive brain stimulation techniques are underway (NCT03826030). Further studies are needed to develop biomarkers to identify the best responders and to guide concomitant interventions to optimize the rehabilitation of stroke patients.

### 3.2. Robot-Assisted Therapies

Robot-assisted therapies (RAT) are another novel modality in which patients are provided upper or lower limb therapy by robotic devices rather than conventional hands-on therapy. RAT can provide a high-intensity standardized therapy, which is thought to be important in motor cortex reorganization and promoting recovery. RAT aims to increase the capacity of motor control of the paretic arm or leg, muscle strength, and upper limb capacity, and thus promote basic activities of daily living.

As a proof-of-concept study, Takahashi and colleagues conducted a 3 week-long upper limb RAT in 13 patients with stroke. They showed that RAT produced a significant motor gain in patients with moderate post-stroke motor deficits [86]. In a larger-scale, multicenter, randomized, and controlled trial, Lo and colleagues compared RAT, intensive comparison therapy, and usual care in chronic stroke patients with moderate-to-severe upper-limb impairments [87]. They showed that RAT increased motor function in 36 weeks compared to standard therapy. However, RAT was not superior to intensive therapy, which suggested that RAT was as good as intensive behavioral therapy. Similarly, Rodgers and colleagues showed that compared with usual care, RAT did not lead to better motor recovery in patients with stroke-related moderate to severe upper limb dysfunction [77]. At this point, RAT is safe and induces positive effects in motor recovery after stroke, but its superiority to usual intense therapy is yet to be proven [88]. Overall, repetition and intensity are the key factors for favorable motor recovery, and RAT is a promising alternative to conventional therapies to achieve these goals.

### 3.3. Telerehabilitation

Telerehabilitation is a method of delivering rehabilitation services remotely using communication methods. This therapy aims to address the main challenges of providing higher doses of rehabilitation therapies, such as cost-related issues, traveling difficulties, limited access to high-quality rehabilitation centers/providers, poor compliance, and dose limitations of in-clinic therapy sessions. Furthermore, telerehabilitation’s principles of high intensity, easy access, and gamification of tasks help to promote a higher quality of therapy by creating more challenging, motivating, and variable goal-oriented tasks in more relevant environments with the supervision and feedback of therapists. A randomized, multicentered, non-inferiority trial showed comparable efficacy of home-based telerehabilitation to traditional in-clinic rehabilitation, with excellent adherence and higher arm movement repetition (average 1031 repetitions per day) [78].

Most studies enrolled patients with chronic stroke patients, but a recent study assessing the optimal time for motor recovery showed that the task-specific motor therapies were most effective within the first 2–3 months, suggesting enhanced neural plasticity in earlier stages after stroke [89]. In clinical practice, the early initiation of rehabilitation is challenging due to limited, fragmented, and often delayed transition care from initial hospitalization to in-facility rehabilitation and/or to home settings. Telerehabilitation may address these challenges of transition and improve earlier access to rehabilitation with better continuity of care. A recent feasibility study supports that telerehabilitation is feasible, safe, and possibly efficacious in providing therapy to early stroke patients [90]. Further controlled studies are needed to expand the use of telerehabilitation.

## 4. Brain–Computer Interfaces

The activity-based therapies described above require some residual movement of the affected limb; however, not all stroke survivors qualify for these treatments. The brain–computer interface (BCI) is an emerging field, especially in settings of technological advances such as virtual reality, robotics, and sensors. BCIs allow control of robotics devices by translating the brain’s neural and/or physiological activity into a signal, completely bypassing the lesion [91]. While BCIs can be used as assistive technology to help with patients’ functions and daily living activities [92], here we focus on their use as rehabilitation technologies. The rehabilitative (also referred to as neurofeedback) BCIs aim to recognize patients’ intention of movement/task with brain signals captured with scalp-recorded electroencephalogram (EEG), then provide a user perceivable feedback using a feedback mechanism (combination of visual displays, robotic devices, exoskeletons, etc.) [93,94]. The aim of BCIs is to enhance neuroplasticity with motor imagery and sensory feedback by closing the gap in the motor intention, execution, and feedback loop.

Most studies assessed rehabilitative BCIs for upper extremity motor recovery. Two metanalyses showed statistically and clinically significant improvement in upper extremity motor functions, measured mainly by the Fugl-Meyer assessment, with effect sizes comparable to other treatment modalities used in stroke rehabilitation [95,96]. There is limited evidence for the use of rehabilitative BCIs for lower extremity recovery; however, it is far less studied due to difficulties in decoding algorithms of lower extremity movement kinematics using non-invasive recordings [97]. The literature is promising for BCIs’ use for cognitive and speech rehabilitation in other brain disorders like cognitive impairments, attention deficit disorders, and traumatic brain injury [98,99,100]. However, currently the data are very limited for post-stroke rehabilitation. Lesion bypass mechanisms with neurofeedback are promising for stroke recovery, particularly with severe post-stroke deficits; further large controlled clinical trials are needed to validate its use in motor recovery and explore its effects in cognitive and speech recovery.

## 5. Pharmacological and Cellular Therapies

Over the past decades, the research on pharmacological therapies primarily focused on neuroprotection in the acute stroke phase; however, many drugs have been studied to possibly enhance stroke recovery through neuroplasticity and neuronal regrowth. Several therapies include anti-depressants, stimulants, dopamine agonists, niacin, memantine, growth factors, monoclonal antibodies, and stem cells. In this review, we focused on promising therapies with translational potentials: selective serotonin reuptake inhibitors, maraviroc, and stem cell therapies.

### 5.1. Selective Serotonin Reuptake Inhibitors (SSRIs)

SSRIs are the most extensively studied pharmacological agent in stroke recovery. Post-stroke depression is common, with higher vulnerability in the early chronic period. Post-stroke depression may directly affect motivation and adherence to participate in rehabilitation. Therefore, identifying and treating depression was of interest to support recovery. However, SSRIs may also enhance synaptic plasticity after an ischemic brain injury besides the anti-depressant effects. This was initially tested with the FLAME (Fluoxetine for Motor Recovery After Acute Ischemic Stroke) trial, which showed a statistically significant improvement in motor recovery, measured by a Fugl-Meyer motor score, in stroke patients treated with fluoxetine [101]. However, a subsequent Cochrane systematic review, including 52 randomized trials, challenged these results, given the methodological limitations and heterogeneity of studies [102]. Therefore, three additional large randomized controlled clinical trials (AFFINITY: Assessment of Fluoxetine in Stroke Recovery, FOCUS: Fluoxetine or Control Under Supervision trials, and EFFECTS: Efficacy of Fluoxetine—a Randomised Controlled Trial in Stroke) were conducted and they did not show functional improvement at 6 months, measured by a modified Rankin Scale (mRS) [103,104,105]. The main issues with these trials include the heterogeneous study population with mainly mild stroke patients, which might decrease the sensitivity to detect group differences. Another limitation was the use of mRS, which may limit the interpretation of these trials. Despite the fact that mRS is commonly used in studies for stroke outcomes to assess functional independence, this measure is potentially insensitive in capturing various improvements in different systems such as motor, language, cognition, etc. SSRIs may still play an important role in treating post-stroke depression and possibly improve recovery, especially if paired with other therapies. Finally, given language recovery was limitedly assessed in these trials, another multicentered, randomized, placebo-controlled phase II trial (ELISA: Escitalopram and Language Intervention for Subacute Aphasia trial) is currently in the recruitment phase, assessing the effect of escitalopram as an adjunct to traditional speech and language treatment [106].

### 5.2. Maraviroc

C-C chemokine receptor 5 (CCR5), which plays a role in learning, memory, and plasticity [107], was recently shown to be differentially upregulated in neurons post-stroke [108]. This study showed that the knockdown of CCR5 in the premotor cortex induces motor recovery after stroke. An FDA-approved drug for the treatment of HIV, maraviroc, has known effects on selectively antagonizing CCR5 function, and this treatment also showed similar effects in recovery in this animal study. Interestingly, they performed an observational study in a large cohort of stroke patients and showed that patients with CCR5 loss-of-function mutation had better recovery after stroke [108,109]. Given these promising results, two randomized controlled phase II trials for human stroke recovery are currently ongoing (NCT03172026, NCT04789616).

### 5.3. Stem Cell Therapies

Current stroke recovery treatments focus on enhancing neuroplasticity but are still limited in terms of their regenerative benefits. Stem cell therapies have been a promising treatment approach for potential neuroprotection and neuroregeneration [110]. Neuroprotective use of stem cells aims to limit the extent of initial damage during the acute stroke phase and expand the window of available treatments. Neuroregenerative stem cell therapies aim to reduce the neuronal loss and to replace injured neurons. Here, we highlight the evidence for the neuroregenerative use of stem cells to enhance post-stroke recovery. The initial evidence is from animal stroke models: one meta-analysis including 141 pre-clinical studies showed improvement in functional outcomes including motor, sensorimotor and cognitive function, without any differences based on delivery route or dose [111]. A phase I/II non-controlled study showed that single-dose intravenous allogeneic mesenchymal stem cells were safe in patients with chronic stroke and substantial functional, and furthermore suggested behavioral gains [112]. A single-center, open-label randomized clinical trial (ISIS-HERMES trial) showed improved motor outcomes after intravenous autologous mesenchymal stem cell combined with rehabilitation [113]. The treatment group had greater motor cortex activation suggesting that this effect was through sensorimotor neuroplasticity. On the other hand, the RECOVERY trial, a phase 2, randomized, sham-controlled study, assessed the safety and efficacy of intracarotid infusion of autologous bone marrow-derived ALD-401 and showed no significant difference in mRS at 90 days [114]. However, there was an increased incidence of smaller lesions in the treatment group. Finally, the PISCES-II trial, an industry-funded, single-arm study, assessed intracerebral CTX0E03 human neural stem cell injection in stroke patients with severe arm motor deficits [115]. Only 1/23 patients met prespecified criteria for responders, 3/23 at 6 and 12 months, and responders were only the ones with residual upper limb movement at baseline. All these studies found that these interventions were safe and feasible. Current studies vary in their protocols with different cell types, delivery methods, and doses; further studies with larger sample sizes and robust methodologies are needed.

## 6. Cognitive-Based Therapies

### 6.1. Prism Adaptation Therapy

Spatial neglect is described as failure to orient, perceive, and/or respond to left space after a right brain stroke, which is accompanied by functional disability and is usually under-recognized [116]. This condition is particularly important for stroke recovery given the challenges such as prolonged hospitalization, requiring more caregiver/supervision, poor motor recovery, and increased fall risk. Despite several therapeutic approaches available for visual perception deficits, such as visual scanning training [117,118], these approaches focus on abnormal visuospatial perception, also defined as “where” spatial neglect [119]. While neglect can present as the classic visual perceptual phenomenon (“where” spatial neglect), it can be in the form of spatial motor-intentional deficits (“aiming” spatial neglect). Aiming spatial neglect can present as postural imbalance, veering while ambulating or in a wheelchair, and out of proportion weakness to motor dysfunction [120]. These deficits are often undetected and may not respond to visual perceptual therapies, limiting their recovery after stroke. Prism adaptation therapy is a novel approach, targeting impairments in spatial motor aiming spatial neglect. 

PAT is a simple and inexpensive therapy that requires stroke patients to wear wedge prism lenses, shifting the visual field approximately 12 degrees rightwards during intensive motor training. This therapy is brief (20 min), easy to perform, and has advantages for in-hospital/clinic use standardization. PAT is well tolerated; adverse effects include mild and transient discomfort or dizziness [121]. Level A evidence supports using cognitive rehabilitation for spatial neglect, including PAT [122]. A systematic review including 26 controlled studies suggests improvement in functional disability during daily living activities with PAT [123]. However, the results from controlled studies are inconsistent and present variability in patient responses to PAT. One of the issues with the current literature is the failure to stratify spatial neglect patients; as discussed above, PAT may be more effective in “aiming” spatial neglect rather than early perceptual deficits. Studies demonstrated specific alterations in spatial motor aiming bias in healthy and stroke patients with PAT [124]. Furthermore, the outcome measure choices in the literature are more oriented to spatial attention or awareness, therefore failing to capture the improvements with PAT [121]. The dosage of PAT is also variable in studies, and a recent study predicted greater improvement with a higher dose of PAT [125]. Finally, the neuroanatomy of stroke lesions may affect the response to PAT. A recent randomized controlled study showed that only patients with frontal lesions improved their neglect symptoms, while the presence of frontal lesions did not affect the response in the standard-care group [126].

Further studies are needed with better classification and stratification of spatial neglect patients, possibly screening for spatial “aiming” deficits to optimize patient selection, better outcome measures including both mechanistic and functional outcomes, and identifying neuroimaging biomarkers for PAT response. Currently, two studies are ongoing to address these questions (NCT05983185, NCT00989430).

### 6.2. Virtual Reality

Virtual reality (VR) and interactive video gaming are other developing treatment approaches in stroke rehabilitation. VR therapies use an interactive simulation where patients/users have the opportunity to engage in an environment similar to the real-world using controllers [127]. Currently, there are three main virtual reality systems; (i) non-immersive, (ii) semi-immersive, and (iii) fully immersive simulations. The non-immersive systems allow a two-dimensional computer-generated environment through consoles and computer displays, where patients can control an avatar using a control tool, similar to common video games. While semi-immersive systems provide a three-dimensional environment with a fixed visual perspective, the fully immersive systems allow patients to experience the most realistic simulation environment where they can immerse themselves using a head-mounted display and extensive motion sensors.

VR offers many advantages in stroke rehabilitation. The duration and repetition (dose) of training are important for recovery; VR can increase the rehabilitation dosage by enabling patients to practice functional tasks in simulated environments. Several studies suggested the feasibility of high-dose rehabilitation using VR [128,129]. The simulated environments also help the effectiveness of sessions by increasing the active training time and, furthermore, can decrease the travel and accessibility-related difficulties. Given the significant variability in study protocols, it is hard to draw definitive conclusions from the existing literature. A meta-analysis in 2017 with 72 studies failed to show significant improvement in upper extremity function of VR compared to conventional therapy, but it was significant when combined with usual care [130,131]. More recent studies since this meta-analysis had positive results, which were particularly attributed to higher doses of therapy [130,131]. The studies with significant improvement had more intensive therapies, suggesting a dose–effect relationship [132]. Besides the dose differences, multiple different VR systems and controllers, as well as newer features including hand movement trackers, enhanced feedback systems, and sensorized hand-held objects, varied widely in the studies. Finally, a recent meta-analysis comparing immersive and non-immersive virtual reality for upper extremity motor recovery suggested superior effects with immersive virtual reality systems [133]. The superior effects of immersive systems could be attributed to higher user perception and sensory feedback, and facilitation of patients to engage in task-oriented activities [134]. Current evidence suggests the beneficial effects of virtual reality, especially with immersive systems, in upper extremity motor recovery. Further studies should focus on providing high-intensity VR therapy and test if using these physical objects or enhanced feedback systems may improve recovery.

### 6.3. Motor Imagery and Action Observation Therapies

Motor imagery and action observation therapies are forms of cognitive treatment that aim to activate motor cortex plasticity without an actual motor execution. While action observation therapy is defined as the perception of other individuals performing a motor task, motor imagery is a mental practice to simulate a movement without motor input [135]. This mental practice theory for neurorehabilitation is based on multiple forms of evidence which indicate that imagery of a motor task induces a similar pattern of brain activity compared to actual motor execution [136]. More recently, the focus of research has been combining motor imagery and action observation, and this combination was found to involve a greater neuronal activity including other supplemental cortical and subcortical motor areas [137]. Therefore, the possible benefits of these treatments have been tested for motor recovery after stroke. Despite motor imagery having promising early studies [138,139], the overall literature is conflicting [140,141]. A meta-analysis showed significant heterogeneity in methodological quality in these trials, and despite detecting a signal for the efficacy of motor imagery for upper and lower extremity motor function and balance, there was no significant difference when only high-quality studies were included [142]. The action observation had more consistent positive effects, especially if combined with concomitant physical therapy of observed action [143,144]. Finally, combining observation and motor imagery had better functional outcomes [145]; however, this needs to be tested further with a larger sample size [146]. Currently, the evidence of these mental practices is limited to drawing a final conclusion; the most promising effect is likely when action observation and motor imagery are combined and when introduced concomitantly with actual physical training.

## 7. Conclusions

Various new approaches and technological advances had promising results in promoting recovery after stroke. These therapies target to enhance neural plasticity and help overcome challenges of rehabilitation access in order to provide an adequate dose of rehabilitation and improve adherence and participation. CIMT and telerehabilitation are promising to overcome these challenges of accessibility and dosing, and the future of recovery is focused on involving virtual reality in activity-based treatments. The multidisciplinary treatment models are crucial for post-stroke neurorehabilitation; further adjuvant treatments with brain stimulation techniques and pharmacological agents should be considered to maximize the recovery. Finally, there is a significant gap in identifying and treating cognitive disorders after stroke, further diagnostic/screening tools and treatment options are needed. One of the main challenges in the field is the methodological variations and heterogeneous patient populations that limit providing an overarching conclusion. The studies and their mixed results point out the importance of matching the appropriate patient population; therefore, developing biomarkers is crucial to guide future research designs and clinical practices. 

## Figures and Tables

**Table 1:** Prominent clinical trials in neuromodulation. UEFM: Upper Extremity Fugl-Meyer; WMFT: Wolf Motor Function Test; MAL: Motor Activity Log; SIS: Stroke Impact Scale; BDI: Beck’s Depression Inventory; Euro-Qol-5D: EQ 5 Dimensions Quality of Life, SS-QOL: Stroke Specific Quality Of Life Scale; ARAT: Action Research Arm Test.

Author, Year	Time Since Stroke	Sample Size (Active/Sham)	Neuromodulation Intervention	Behavioral Intervention and Frequency	Outcome Metrics	Main Efficacy Outcomes
Dawson, 2021 [7]	9 months–10 years	53/55	VNS	Standardized 7-tasks arm in-clinic training (18 sessions over 6 weeks) followed by home exercise	UEFM, WMFT, MAL, SIS, BDI, Euro-Qol-5D, SS-QOL	Clinically meaningful response (UEFM > 6 points) was seen in 47% in active vs. 24% in sham group (*p* < 0.01) at 90 days
Fridrikson, 2018 [8]	>6 months	34/40	tDCS	Computerized speech therapy	Correct Naming on Philadelphia Naming Test and 80 Trained Items	Relative 70% increase in correct naming for A-tDCS relative to sham and no futility.
Harvey, 2018 [9]	3 to 12 months	132/67	rTMS	Task-oriented upper limb therapy (18 sessions over 6 weeks)	UEFM, WMFT, ARAT, SIS Euro-Qol-5D, PHQ-9	Both groups had clinically meaningful response, without significant difference (*p* = 0.76)
Vink, 2023 [10]	Within 3 weeks	29/31	cTBS	Standard upper limb therapy (10 sessions)	ARAT, UEFM, SIS, Euro-Qol-5D	Significant improvement in active cTBS (9.6 points in ARAT, *p* = 0.0244)
Stockbridge 2023 [11]	<3 months	30/28	tDCS	Computer-delivered naming treatment	Philadelphia Naming Test	No significant difference (*p* = 0.54)

**Table 2:** Prominent clinical trials in activity-based interventions. WMFT: Wolf Motor Function Test; MAL: Motor Activity Log; ARAT: Action Research Arm Test; UEFM: Upper Extremity Fugl-Meyer.

Author, Year	Time Since Stroke	Sample Size (Active/Control)	Study Intervention	Control Group	Outcome Metrics	Main Safety and Efficacy Outcomes
Wolf, 2006 [76]	3 to 9 months	106/116	Constraint-induced movement therapy	Usual care	WMFT, MAL	Significant reduction in WMFT performance time (*p* < 0.001)
Rodgers, 2019 [77]	1 week to 5 years	257/259/254	Robot-assisted training or enhanced upper limb therapy	Usual care	ARAT	No significant difference
Cramer, 2019 [78]	4 to 36 weeks	62/62	Telerehabilitation	In-clinic therapy	UEFM	Non-inferiority of telerehabilitation
